# Assessing the impact of screening, brief intervention, and referral to treatment implementation on attitudes toward and moralization about alcohol, tobacco, and other drug use

**DOI:** 10.1186/1940-0640-7-S1-A69

**Published:** 2012-10-09

**Authors:** Telmo Ronzani, Marina Oliveira, Daniela Mota, Erica Cruvinel, Tamires Laport, Leonardo Martins

**Affiliations:** 1 Department of Psychology, Federal University of Juiz de Fora, Juiz de Fora, Brazil; 2 Department of Social Psychology and Public Health, Federal University of Juiz de Fora, Juiz de Fora, Brazil

## 

Screening, brief intervention, and referral to treatment (SBIRT) dissemination has been presented as a way to change medical professionals’ attitudes toward adopting prevention practices for alcohol and other drug use. We evaluated the impact of SBIRT dissemination on attitudes related to preventive practices for alcohol consumption and on the moralization of alcohol and other drug use by primary care professionals (N = 123) in two Brazilian cities. Eighty-two participants received SBIRT training (intervention group), while 41 received no SBIRT training (control group). At baseline and three-month follow-up, both groups completed scales of beliefs and attitudes regarding SBIRT implementation and another scale about the moralization of tobacco, alcohol, marijuana, cocaine, and crack use. The intervention group showed an increase in the perception of obstacles to screening implementation at follow-up assessment (p < 0.05). Regarding moralization, the intervention group had lower moralization for crack consumption than controls at follow-up (p < 0.05). The other measures showed no significant differences. According to these results, SBIRT training and three months of practice supervision were not sufficient to promote changes in primary care professionals’ attitudes toward drug and alcohol screening or to reduce substance use moralization. These findings are relevant to research related to SBIRT dissemination, since merely learning SBIRT techniques without attitudinal changes and moralization reduction compromises the quality and effectiveness of those practices with regard to alcohol and other drug use.

